# An antisense amido-bridged nucleic acid gapmer oligonucleotide targeting *SRRM4* alters *REST* splicing and exhibits anti-tumor effects in small cell lung cancer and prostate cancer cells

**DOI:** 10.1186/s12935-022-02842-1

**Published:** 2023-01-17

**Authors:** Misa Yoshida, Chihiro Oda, Keishiro Mishima, Itsuki Tsuji, Satoshi Obika, Masahito Shimojo

**Affiliations:** 1 Graduate School of Pharmaceutical Sciences, Osaka University, 1-6 Yamadaoka, Suita, Osaka 565-0871 Japan; 2 School of Pharmaceutical Sciences, Osaka University, 1-6 Yamadaoka, Suita, Osaka 565-0871 Japan; 3 Institute for Open and Transdisciplinary Research Initiatives (OTRI), Osaka University, Osaka, 565-0871 Japan; 4 National Institutes of Biomedical Innovation, Health and Nutrition (NIBIOHN), Ibaraki, Osaka 567-0085 Japan

**Keywords:** REST/NRSF, Small cell lung cancer, SCLC, Antisense oligonucleotide, Gapmer, SRRM4, Prostate cancer

## Abstract

**Background:**

Antisense oligonucleotide (ASO) medicine for clinical applications has been becoming a reality. We previously developed a gapmer ASO targeting Ser/Arg repetitive matrix 4 (SRRM4) that is abnormally expressed in small cell lung cancer (SCLC). However the detailed mechanism of ASO through repressing SRRM4 has not been completely elucidated. Further, effectiveness of SRRM4 ASO to prostate cancer (PCa) cells expressing SRRM4 similar to SCLC remains to be elucidated. RE1-silencing transcription factor (REST) is a tumor suppressor, and its splicing isoform (sREST) is abnormally expressed by SRRM4 and causes carcinogenesis with neuroendocrine phenotype in SCLC. The present study aimed to understand the contribution of *REST* splicing by SRRM4 ASO administration.

**Methods:**

SRRM4 expression and REST splicing were analyzed by RT-qPCR and conventional RT-PCR after treating SRRM4 ASO, and cell viability was analyzed in vitro. Exogenous reconstitution of Flag-tagged REST plasmid in SCLC cells and the splice-switching oligonucleotide (SSO) specific for REST was analyzed for cell viability. Furthermore, we expanded the application of SRRM4 ASO in PCa cells abnormally expressing SRRM4 mRNA in vitro.

**Results:**

SRRM4 ASO successfully downregulated SRRM4 expression, followed by repressed cell viability of SCLC and PCa cells in a dose-dependent manner. Administration of SRRM4 ASO then modified the alternative splicing of *REST*, resulting reduced cell viability. REST SSO specifically modified REST splicing increased REST expression, resulting in reduced cell viability.

**Conclusions:**

Our data demonstrate that a gapmer ASO targeting SRRM4 (SRRM4 ASO) reduces cell viability through splicing changes of *REST,* followed by affecting REST-controlled genes in recalcitrant tumors SCLC and PCa cells.

**Supplementary Information:**

The online version contains supplementary material available at 10.1186/s12935-022-02842-1.

## Background

Antisense oligonucleotide (ASO) medicine is currently an effective treatment for intractable diseases [1]. Although several clinical trials are being conducted, anti-tumor drugs have not yet emerged as therapeutic treatments. We previously developed ASO targeting Ser/Arg repetitive matrix 4 (SRRM4), which has a gapmer structure [2, 3]. The gapmer ASO contains a central block of DNA with a wing region of our artificial amido-bridged nucleic acids (AmNAs) [4], Additional file 1: Fig. S1 exhibiting higher affinity for its target SRRM4 mRNA. SRRM4 ASO is a single-stranded oligonucleotide that specifically binds target SRRM4 mRNA sequences that inhibit SRRM4 expression through inducing mRNA degradation by RNase H [3, 5]. ASO containing AmNAs is a phosphorothioate-linked structure with higher affinity to target sequence, nuclease resistance, and low toxicity [4]. Patients with small cell lung cancer (SCLC) express abnormally high levels of RNase H [6], suggesting that the application of an ASO is a good therapeutic strategy.

Lung cancer has a high mortality rate and is classified into the two following types: SCLC and non-SCLC (NSCLC). SCLC is a high-grade neuroendocrine carcinoma that is associated with air pollution and tobacco smoking. SCLC has spread quickly and widely, resulting in an extremely high mortality rate. More than 250,000 people die of SCLC annually worldwide [7]. SCLC is a refractory cancer that easily acquires resistance to drugs. There has been a distinct paucity of significant breakthroughs in SCLC therapy over the last 30 years, SCLC has been classified as a recalcitrant cancer [8].

Difficulties in clinical treatment is due to the combination of SCLC and NSCLC as well as the transformation between SCLC and NSCLC. For patients with NSCLC, several effective molecular target medicines are available. However, even patients with NSCLC frequently also harbor SCLC cell types, resulting in a deadly prognosis. Tumor heterogeneity of SCLC and NSCLC is caused by several pathways [6, 9]. An important key molecule is the transcriptional repressor RE1-silencing transcription factor (REST), also known as neuron-restrictive silencer factor. The neuroendocrine phenotype of SCLC is caused by the expression of neuronal genes that are controlled by the master molecule REST [10]. REST is a transcription repressor that targets most neuronal genes [11]. In general, REST is highly expressed in non-neuronal cells, thereby suppressing RE1-controlled genes [12]. Dysregulation of REST can be observed in the lung cancer as well as in aggressive prostate cancer (PCa) [13]. In SCLC, abnormal expression of the splicing variant of REST (sREST) has been reported [14]. REST specifically binds the RE1 sequence of target genes through its zinc finger domains, resulting in the repression of RE1-controlled genes, while sREST is truncated form of REST missing of important zinc finger domains. The expression of sREST competes with REST binding to RE1 genes, permitting abnormal expression of target genes, resulting in a loss of tumor repressor [15]. REST is highly expressed in stem cells as well as non-neural cells [12], and it mediates the cell differentiation of SCLC and PCa [16–18]. Thus, dysregulation of *REST* resulting in suppression of RE1-controlled genes causes neuroendocrine phenotypes to appear. Therefore, the dysregulation of alternative splicing induces the progression of tumors to an aggressive phenotype in SCLC and PCa [19]. A key factor regulating the alternative splicing of *REST* is SRRM4.

SRRM4 is a novel splicing activator specifically expressed in the normal brain, which induces the splicing of REST to its non-functional isoform sREST [20]. Abnormal sREST is highly expressed in SCLC, which correlates with its phenotype with the expression of several neuronal markers [16, 17]. Abnormal expression of SRRM4, which causes the loss of REST activity through alternative splicing to sREST, promotes the emergence of neuroendocrine phenotypes of cancers [21]. Most recently, the splicing program controlled by SRRM4 was shown to play a role in cancer proliferation and differentiation [22]. Thus, *SRRM4* was selected as a therapeutic target for the neuroendocrine tumor SCLC as well as PCa.

Administration of SRRM4 ASO we previously developed effectively exhibited anti-tumor effects both in vitro and in vivo [3], while the detailed mechanism has not been well-studied. Here, we report that the administration of SRRM4 ASO causes anti-tumor effects through the modification of alternative splicing from sREST to REST.

## Methods

### Cell culture

All cell lines were obtained from the American Type Culture Collection (ATCC). All SCLC cell lines were cultured as floating aggregates, and PCa cell lines as adherent cells grown in RPMI-1640 medium (FUJIFILM Wako Pure Chemical, #187-02705) containing 10% FBS (Thermo Fisher Scientific, #10270106). Cells were cultured at 37 °C in a humidified incubator with 5% CO_2_. The cell lines used were as follows: SCLC cell lines, NCI-H146 (HTB-173), NCI-N417 (CRL-5809), and NCI-H209 (HTB-172); PCa cell lines, 22Rv1 (CRL-2505) and VCaP (CRL-2876).

### In vitro transfection of ASOs

For transfecting floating SCLC cells, cells (0.5 × 10^6^) were transfected using the Neon Transfection System according to the manufacturer’s instructions (Thermo Fisher Scientific) and cultured in 2 ml of culture medium. Briefly, various amount of SRRM4 ASO (nmol/0.5 × 10^6^ SCLC cells or nmol/0.4 × 10^6^ for PCa cells) shown in each figure were used in 10 or 100 µl in tip for electroporation system. The conditions are pulse voltage: 1200 v, pulse width: 20 ms, pulse number: 2 pulses. For adherent cells, transfection to 22Rv1 and VCaP cells (confluence of 70–80%) using Lipofectamine3000 was performed according to the manufacturer’s instructions (Thermo Fischer Scientific). Briefly, cells (0.4 × 10^6^/well) on 6-well plate were incubated with 7.5 l of Lipofectamine3000 in 2 ml medium with various amount of SRRM4 ASO shown in each figure. Phosphorothioate ASO (AmNA_7174) targeting SRRM4 mRNA was synthesized and purified by Gene Design, Inc. (Osaka, Japan). The sequences were 5’-TGAACAAAATAATAC-3’ (AmNA_26) and 5’-TTGTGTGACTGAAGC-3’ (AmNA_7174). Nucleotides underlined are artificial nucleotides with amido-bridged nucleic acids (AmNAs).

### Reverse transcription-quantitative polymerase chain reaction (RT-qPCR)

Total RNA was prepared according to the RNeasy Plus Micro Kit according to the manufacturer’s instructions (Qiagen). Total RNA was quantified spectrophotometrically at 260 nm and verified to be of high quality. RNA quality of some samples prepared in same methodology was assessed by Bioanalyzer (Agilent), in which RNA integrity number (RIN) were mostly > 7.0 in several RNA preparation. Total RNA (100 or 200 ng) was transcribed at 42 °C for 60 min using the SuperScript VILO kit (Thermo Fischer Scientific). Amplification was conducted using a StepOnePlus Real-Time PCR System (Thermo Fisher Scientific). For SRRM4, forward and reverse primers (10 pmol each) were used in 20 μl reactions. For β-actin, the master mix was made using TaqMan β-Actin Detection Reagents (Thermo Fisher Scientific, #401846). Each master mix was made using TaqMan Fast Advanced Master Mix (Thermo Fisher Scientific, #4444964). Aliquots of cDNA (1/10 of RT reaction) were analyzed by RT-qPCR. qPCR was carried out with initial activation at 95 °C for 20 s followed by 40 cycles of amplification (95 °C for 3 s, 60 °C for 30 s). Fluorescence development was assayed once per cycle of amplification, as recommended by the manufacturer. Relative mRNA levels were analyzed according to the 2^-ΔΔCt^ method, in which all ΔCt values were normalized to β-actin (actin). All qPCR experiments were carried out in at least triplicate, and the mean ± SD values were calculated. PCR products were analyzed as single bands on agarose gels and by DNA sequencing to confirm the products. The primer sequences were as follows: hSRRM4 forward: 5ʹ-tgacaaagacttgacaccacc-3ʹ; hSRRM4 reverse: 5ʹ-acctgcgtcgcttgtgttt-3ʹ; TaqMan Probe: 5ʹ-FAM-aggtcctcatcctatagcccatcgcct-TAMRA-3ʹ. The primers and probe used were synthesized and obtained from Thermo Fisher Scientific.

### Assessment of cell viability

The viability of the cells was measured using Cell Counting Kit-8 (CCK-8, Dojindo) or CellTiter-Glo 3D cell viability assay (Promega) according to the manufacturer’s protocol. After culturing with or without treatment in a 96-well plate for appropriate culture time, the assay was performed in triplicate more than 3 times to confirm reproducibility. The signal was analyzed using Infinite M1000 (Tecan) or GloMax microplate reader (Promega).

### Western blot analysis

Flag-tagged REST and Flag-tagged sREST (5 or 15 g/1.0 × 10^6^ cells) were transfected in H146 cells in 100 µl in tip with electroporation system. The conditions are pulse voltage: 1200 v, pulse width: 20 ms, pulse number: 2 pulses. After culturing of H146 cells in 2 ml of medium for 24 h, protein lysates were prepared in RIPA buffer according to the manufacturer’s instructions (Nacalai Tesque, #08714-04). Aliquots of total protein were fractionated by SDS-PAGE on a 7.5% Mini-PROTEAN TGX precast gel (Bio-Rad, #4561023) and transferred to a nitrocellulose membrane using Transblot Turbo (Bio-Rad). The membrane was processed using the iBind Automated Western System at room temperature (20–25 °C) according to the manufacturer’s instructions (Thermo Fisher Scientific). The membrane was reacted with anti-Flag antibody (Sigma-Aldrich, #F4042; 1:1000 dilution) and anti-β actin (MBL, #PM053; 1:1000 dilution) and a fluorescent-labeled secondary antibody (Thermo Fisher Scientific #A32729, #A32735; 1:1000 dilution) using iBind Flex Fluorescent Detection (FD) Solution Kit (Thermo Fisher Scientific).

### Reverse transcription-polymerase chain reaction (RT-PCR)

Total RNA (100 or 200 ng) was transcribed at 42 °C for 60 min using the SuperScript VILO kit (Thermo Fischer Scientific). Aliquots of cDNA (1/10 of RT reaction) were amplified using Hot Start Taq DNA Polymerase (New England Biolabs). Amplification was conducted using a PCR Thermal Cycler Dice Touch (TaKaRa). PCR was carried out with initial activation at 95 °C for 30 s followed by 20 (β-actin) or 27 cycles (REST, sREST) of amplification (95 °C for 15 s, 58 °C for 15 s, and 68 °C for 30 s) and 68 °C for 2 min. Forward and reverse primers (5 pmol each) were used in 50 μL reactions. PCR products were analyzed by electrophoresis using 5% Mini-PROTEAN TBE Precast Gels (Bio-Rad), followed by SYBR Gold or EtBr staining (Thermo Fisher Scientific). The primer sequences were as follows: REST forward: 5ʹ- gaacgcccatataaatgtgaa-3ʹ; REST reverse: 5ʹ-tttgaagttgcttctatctgctgt-3ʹ; actin forward: 5ʹ-ggccgtcttcccctccatcg-3ʹ; actin reverse: 5ʹ-ccagttggtgacgatgccgtgc-3ʹ. The number of % exclusion was calculated as the intensities of REST band against total band intensities of REST and sREST.

### Splice-switching oligonucleotide (SSO)

REST SSO was selected after screening using SSO around exon N on the human REST sequence (PCT application is pending. Manuscript is currently prepared for submission.). Briefly, after transfection of SSO in cells followed by RT-PCR, band intensities of REST and sREST were analyzed with iBright Imaging system (Thermo Fisher Scientific). REST SSO is an 18 mer-phosphorothioate oligonucleotide (5ʹ-ATCTAGATCACACTCTAG-3ʹ) with each phosphorothioate linkage containing AmNA nucleosides (underlined, Additional file 1: Fig. S1) in alternating positions with deoxynucleosides within the sequence, starting with 3’-deoxynucleoside. Negative control oligonucleotide (5ʹ-CGCCACAACTATCACTAT-3ʹ) is a scrambled sequence based on other neighboring REST SSO, that does not completely match to human genome database (GGGenome; gggenome.dbcls.jp).

### Microarray analysis

Total RNA (500 ng) was labeled with GeneChip WT Pico Reagent Kit. Samples were then hybridized onto human Genechip Clariom D array, human (Thermo Fisher Scientific). Hybridization images were then scanned and digitized with the Genechip Scanner 3000 (Thermo Fisher Scientific). The normalized signal intensity was log2 transformed, and data analysis was performed with Transcriptome Analysis Console Software (Thermo Fisher Scientific).

### PrimeFlow RNA assay

PrimeFlow RNA assay (Thermo Fisher Scientific) was performed on SCLC cells in culture following the manufacturer’s protocol. Cells were then fixed for 30 min at 4 °C. After permeabilization, cells were fixed a second time for 1 h at RT with Fixation buffer 2. A hybridization step was performed by incubating the cells with the appropriated target probe sets for 2 h at 40 °C. Samples were stored over night at 4 °C in the dark. The day after, pre-amplification and amplification of the hybridization with SRRM4 probe (Thermo Fisher Scientific, #PF-210) was performed by 2 consecutive incubations of 1.5 h at 40 °C with the pre-amplification mix and subsequently the Amplification mix. Finally, cells were incubated with the label probe sets for an hour at 40 °C.

### Immunohistochemistry

Cells were rinsed twice with ice-cold PBS and mounted on slide glass using SmearGell (GenoStaff). The embedded samples were fixed with 4% paraformaldehyde/TBS for 60 min at room temperature. They were then rinsed twice with TBS, and permeabilized with 0.5% Triton X-100 in TBS for 60 min at room temperature. After rinsing twice with TBS, the samples were blocked for 60 min with 10% normal goat serum (Thermo Fisher Scientific), followed by rinsing twice with TBS. Incubate with anti-Ki-67 mouse monoclonal antibody (× 200) and anti-E-cadherin (24E10) antibody (× 200) for 60 min at room temperature. After rinsing twice with TBS, samples were incubated with goat anti-rabbit IgG-Alexa647 and goat anti-mouse IgG-Alexa488 (Thermo Fisher Scientific) (× 500) for 60 min at room temperature. After washing with TBS twice at room temperature, slide glass was mounted with Prolong Gold Antifade mountant with DAPI (Thermo Fisher Scientific). Images were acquired using BZ-X800 (Keyence). All the images were obtained and analyzed in a single setting. The primary antibodies used were Ki-67 (8D5) (Cell Signaling Technology #9449S) and E-cadherin (24E10) (Cell Signaling Technology #3195S).

### Statistical analysis

Assay of each point was analyzed at least triplicate and all of experiments were performed multiple times for confirming reproducibility. Data are basically represented as mean ± standard error of the mean (SEM) of 3 independent experiments. Statistic differences were analyzed using one-way analysis of variance (ANOVA) followed by the Student’s *t*-test. **P < 0.01; ***P < 0.001.

## Results

### Analysis of SRRM4 expression by SRRM4 ASO in SCLC cells

**Fig. 1:**
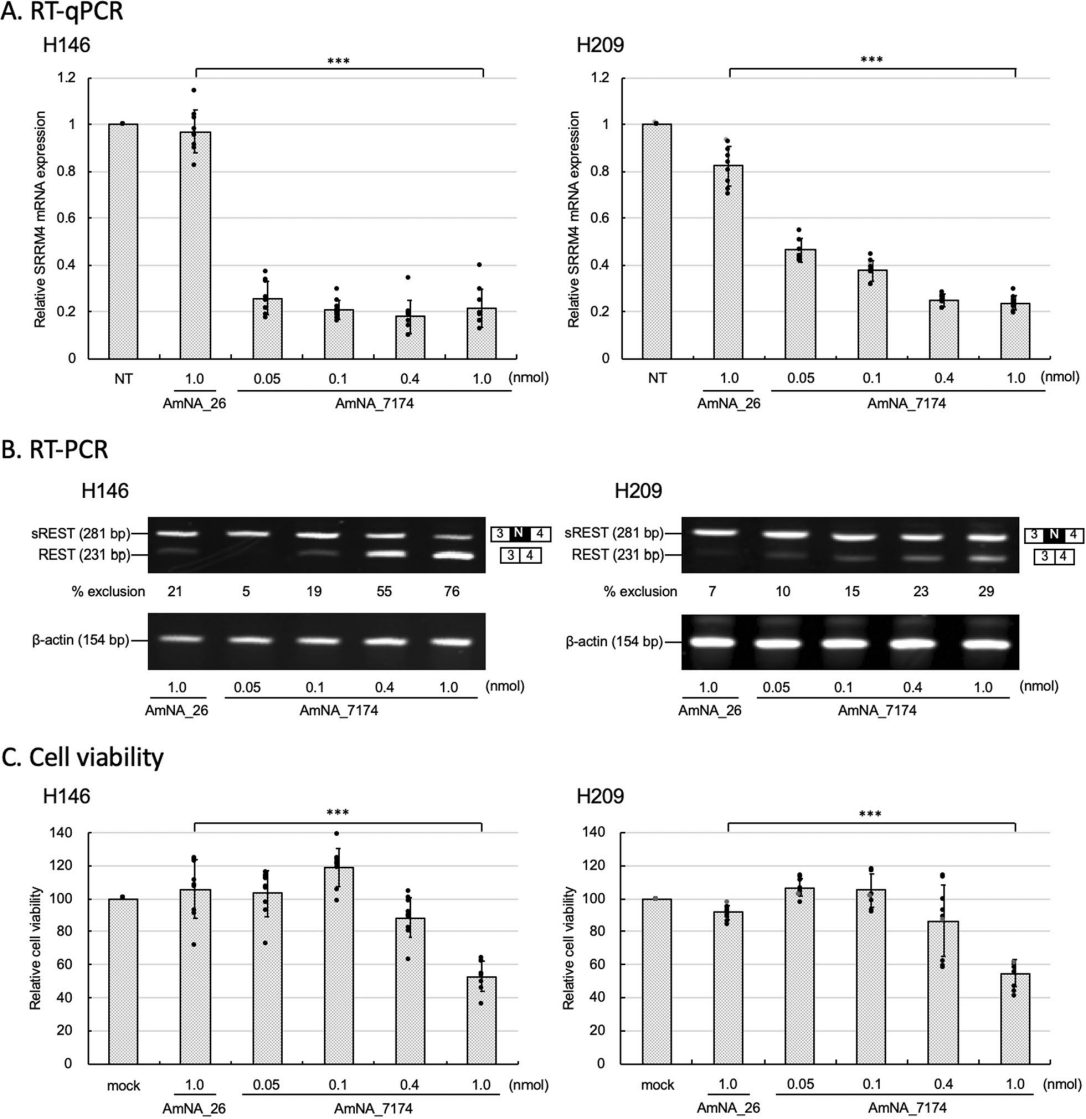
SRRM4 ASO affects the cell viability and promote *REST* splicing change. **a** SRRM4 mRNA expression analysis in SCLC cells transfected with SRRM4 ASO. SCLC cells (H146 and H209) were transfected with various amounts of ASO (AmNA_7174) and negative control oligonucleotide (AmNA_26). Cells (0.5 × 10^6^) were transfected with SRRM4 ASO and collected 24 h after transfection. RT-qPCR was performed using specific primers, and the 2^−ΔΔct^ values of SRRM4 mRNA expression were normalized to endogenous actin in non-treated cells (NT) as 1.0. The assay was performed 3 times to confirm reproducibility. Data are presented as the mean ± SEM of three independent experiments (n = 3). ***P < 0.001. **b** REST splicing was analyzed by RT-PCR. Cells were collected after 72 h following transfection and total mRNA was prepared. RT-PCR was performed using specific primers for either REST or β-actin. PCR products were analyzed by electrophoresis using polyacrylamide gels followed by staining. One of three independent experiments is shown. **c** After 72 h of transfection, cell viability was measured by CellTiter-Glo 3D assay. The assay was performed 3 times to confirm reproducibility. Data are presented as the mean ± SEM of three independent cell cultures (n = 3). Statistic differences were analyzed using one-way analysis of variance (ANOVA) followed by the Student’s *t*-test ***P < 0.001

A previous study showed that gapmer SRRM4 ASO successfully repressed SRRM4 mRNA expression, resulting in SCLC cell death [3]. To expand the therapeutic effects, SRRM4 ASO was transfected with SCLC cell lines H146 and H209. These cell lines were classified into subtype SCLC-A of human SCLC [23]. As most SCLC cell lines are floating cells and it is mostly difficult to transfect ASO, electroporation was initially used for higher transfection efficiency of SRRM4 ASO. Cells transfected ASO were independently grown in separate culture vessels for following each assay. After 24 h of post-transfection, SRRM4 ASO (AmNA_7174) repressed SRRM4 mRNA in a dose-dependent manner (Fig. 1A). The non-specific oligonucleotide AmNA_26 targeting SRRM4 mRNA did not affect SRRM4 expression. AmNA_7174 seemed to be effective at relatively lower concentrations in H146 cells than in H209 cells due to the endogenous expression of SRRM4 (Additional file 1: Fig. S2). *REST* splicing change by SRRM4 ASO might be appeared with delay in time, but it might be due to the endogenous REST mRNA expression.

### Analysis of *REST* splicing by SRRM4 ASO transfection

As SRRM4 is a splicing activator of the tumor suppressor REST, we analyzed the alternative splicing of REST to isoform sREST. In SCLC, REST expression is decreased while sREST expression increases, suggesting that abnormal sREST expression plays a role in the carcinogenesis of SCLC cells [13, 24]. SRRM4 activates the insertion of the neuron-specific exon N which contains stop codon, resulting in the production of an isoform of REST (sREST) (Additional file 1: Fig. S3). To analyze the alternative splicing of *REST*, RT-PCR was performed after transfecting AmNA_7174. The forward primer was set in exon 3 and reverse primer was in exon 4. After PCR, the product containing exon N was 281 bp, while that without exon N was 231 bp (Fig. 1B). Each PCR product was confirmed by sequencing. The data transfecting negative control AmNA_26 showed mainly sREST (281 bp) with a small amount of REST (231 bp). By comparing the band intensities, the % exclusion of exon N was calculated. *REST* splicing changes in H146 and H209 occurred in a dose-dependent manner. The exclusion of exon N by 1.0 nmol of SRRM4 ASO was 76% in H146. The expression (%) of REST mRNA in contrast to sREST mRNA was increased by SRRM4 ASO transfection.

### Analysis of cell viability by SRRM4 ASO transfection

Next, cell viability was analyzed by transfecting SRRM4 ASO (1.0 nmol/0.5 × 10^5^ cells) at 72 h of transfection (Fig. 1C) based on the preliminary time-course of cell viability data (Additional file 1: Fig. S4). Cell viability tended to continue to decrease after 72 h. AmNA_7174 (1.0 nmol/1 × 10^6^ cells) was transfected in H146 and H209 cells, and the cell viability was statistically decreased by approximately more than 60% at 72 h post-transfection. Under the same conditions, SRRM4 ASO did not affect cell viability in NSCLC A549 cells that expressed no SRRM4 mRNA (Additional file 1: Fig. S5).

### Exogenous reconstitution of REST in SCLC cells

**Fig. 2:**
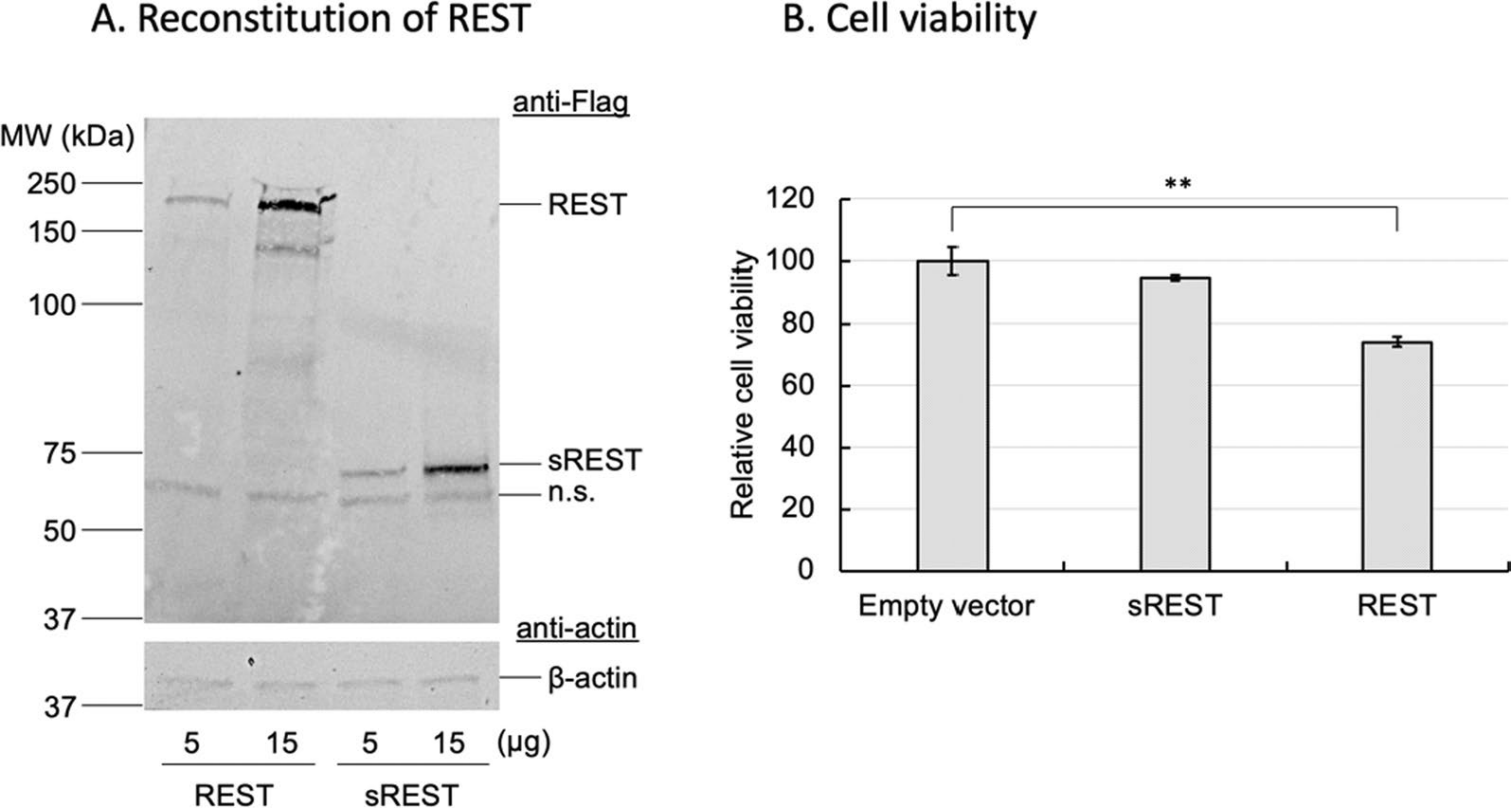
Reconstitution of REST by the exogenous vector in SCLC cells reduced cell viability. **a** SCLC cells (H146) were transfected with each Flag-tagged REST or sREST expression plasmid (5 or 15 μg/1.0 × 10^6^ cells) by electroporation. Then, 24 h post-transfection, cells were collected and cell lysates were prepared. All samples were analyzed by SDS-PAGE followed by immunoblot analysis using anti-Flag antibody. n.s.; non-specific band **b** SCLC cells (H146) were transfected with each plasmid (5.0 μg /1.0 × 10^6^ cells) by electroporation. After 72 h of transfection, cell viability was measured by CCK-8 assay. The assay was performed 3 times to confirm reproducibility. Data are present as the mean ± S.D. in triplicate. **P < 0.01

We assume that SRRM4 ASO represses SRRM4 expression, resulting in modification of *REST* alternative splicing. Thus, we analyzed the effect of the exogenous reconstitution of REST in H146 SCLC cells. Flag-tagged REST and Flag-tagged sREST were previously constructed using pCMV-Tag2 vector [25]. Each Flag-tagged expression plasmid was transfected in H146 cells cultured for 24 h and cell lysates were analyzed for each expression by western blot analysis (Fig. 2A). The single band corresponding each Flag-tagged REST or Flag-tagged sREST without any non-specific bands were appeared confirming the specific expression of each protein. Although data that empty plasmid was not shown for immunoblot, no expression was confirmed even in previous report [25]. After transfection of each plasmid, cell viability of SCLC by the expression of REST, but not sREST, decreased significantly compared to that of the empty plasmid (Fig. 2B). Exogenous expression analysis of REST was repeated several times with increasing amount of plasmid, the result is similar to further reduced cell viability. However further increased amount of plasmid, not only REST plasmid but sREST plasmid caused decreased cell viability. The possibilities might be due to the non-specific toxicity of exogenous expression using higher amount of plasmid, lower transfection efficiency of plasmid and/or intratumoral heterogeneity of SCLC cells [26]. Then we tried to modify directly *REST* splicing to analyze the cell viability.

### Modification of *REST* splicing reduces cell viability in SCLC cells

**Fig. 3:**
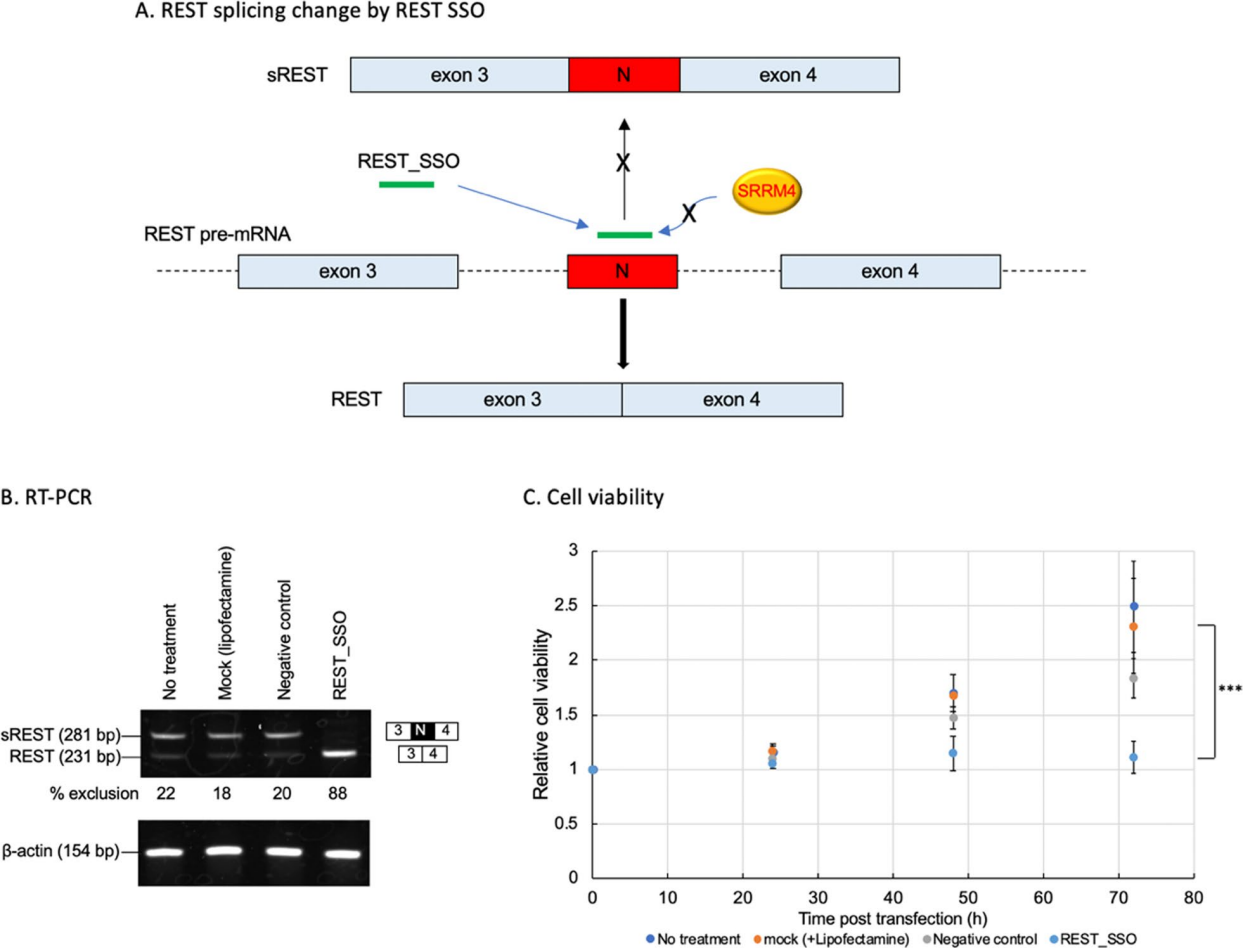
*REST* splicing analysis and cell viability in SCLC cells transfected with REST SSO. **a** Scheme of *REST* splicing change by REST SSO. **b** SCLC cells (H146) were transfected with SSO (AmNA_[+ 21/ + 40]) or negative control oligonucleotide. Cells were collected after 48 h of transfection and total RNA was prepared. RT-PCR was performed using specific primers for *REST* and actin and PCR products were analyzed using polyacrylamide gels; **c** Cell viability was measured by CellTiter-Glo 3D assay every 24 h until 72 h of culturing. The assay was performed 3 times to confirm reproducibility. Data are presented as the mean ± SEM of three independent experiments (n = 3). ***P < 0.001

SRRM4 ASO modified the splicing to produce REST, and exogenous expression of REST reduced cell viability (Figs. 1 and 2). To analyze whether modification of the alternative splicing *REST* directly caused SCLC cell viability, alteration of REST expression by REST splice-switching oligonucleotide (SSO) was performed. We screened for an effective SSO that controls the skipping of exon N in the pre-mRNA of REST and an SSO (AmNA_[+ 21/ + 40]) was selected. REST SSO blocks the binding of splicing factor such as SRRM4 as a concept, resulting the inhibition of exon N inclusion (Fig. 3A). After the transfection of REST SSO in H146 cells, sREST mRNA appeared shifted to REST mRNA judged by RT-PCR, compared to the negative control oligonucleotide. The band of sREST (281 bp) decreased, while that of REST (231 bp) increased completely, but no changed with negative control and lipofectamine (mock) respectively (Fig. 3B). Along with that, cell viability decreased significantly after 72 h of transfection with REST SSO compared to negative control or mock (Fig. 3C).

### SRRM4 ASO affected SRRM4 expression and cell viability in prostate cancer cells

**Fig. 4:**
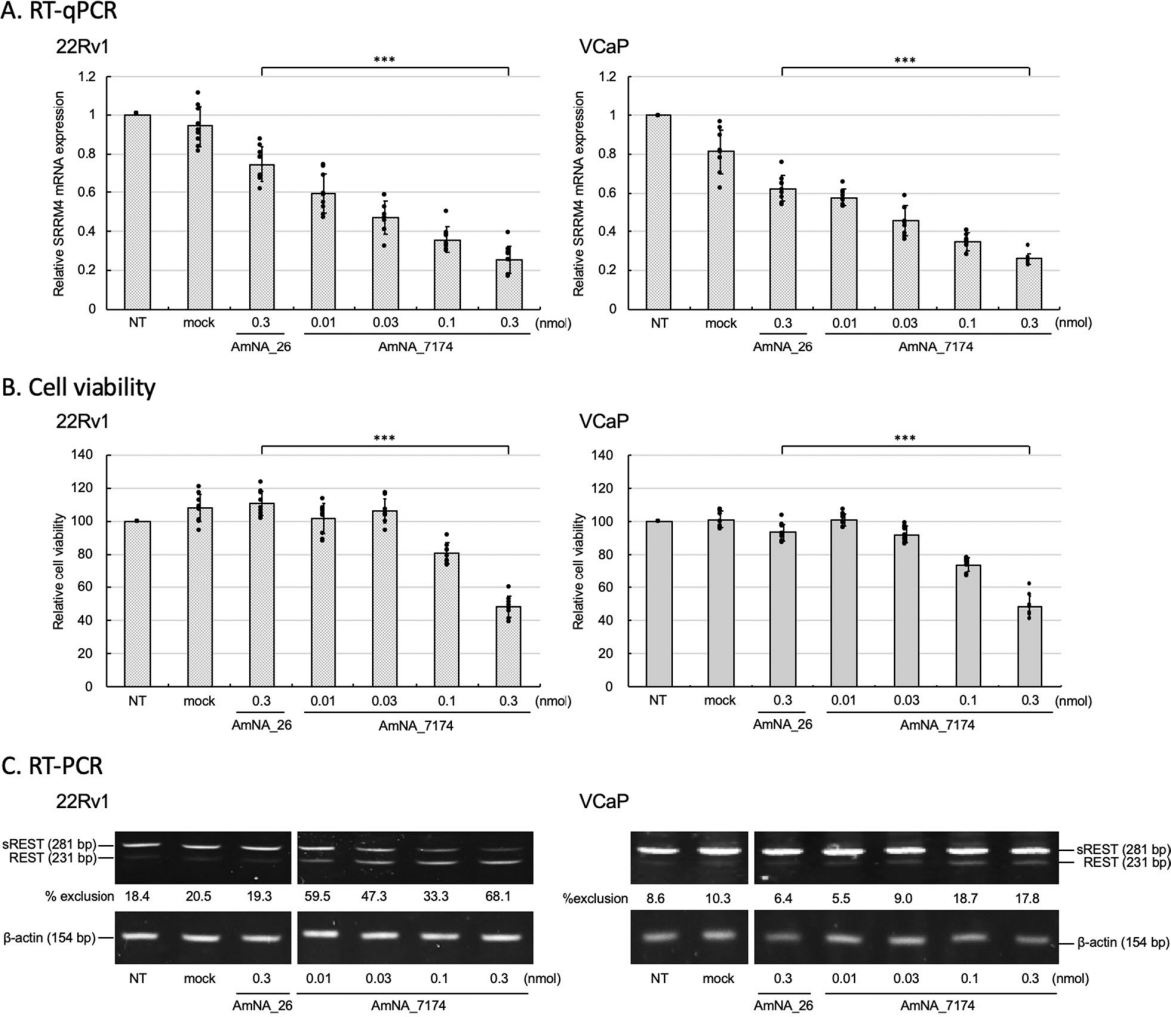
SRRM4 ASO decreased cell viability in SRRM4-expressing prostate cancer (PCa) cells. **a** PCa cells expressing SRRM4 (22Rv1 and VCaP) were transfected with various amounts of ASO (AmNA_7174) and negative control oligonucleotide (AmNA_26). Cells were collected after 24 h following transfection and RT-qPCR was performed using specific primers and 2^−ΔΔct^ values of SRRM4 mRNA expression were normalized with endogenous actin in non-treated cells (NT) as 1.0. The assay was performed multiple times with different amount of AmNA_7174 to confirm reproducibility. Data are presented as the mean ± SEM of three independent experiments (n = 3). ***P < 0.001. (*t* test); **b** After 72 h of transfection, cell viability was measured by CellTiter-Glo 3D assay. The assay was performed multiple times with different amount of AmNA_7174 to confirm reproducibility. Data are presented as the mean ± SEM of three independent experiments (n = 3). ***P < 0.001; **c** Cells were collected after 48 h following transfection. RT-PCR was performed using specific primers for REST and β-actin. PCR products were analyzed by performing electrophoresis using polyacrylamide gels. One of three independent experiments is shown

We next expanded the effectiveness of SRRM4 ASO in SRRM4-expressing PCa cells. After screening SRRM4 mRNA expression in PCa cell lines, we found that PCa cell line VCaP cells expressed SRRM4 mRNA (Additional file 1: Figs. S2 and S7). As VCaP cells as well as other PCa cell line 22Rv1 cells expressing SRRM4 mRNA are adhesive cell lines, we used lipofectamine instead of electroporation for transfection. Each cell line was transfected with various amounts of AmNA_7174 and the negative control AmNA_26. After 24 h of transfection, SRRM4 mRNA was analyzed by RT-qPCR. SRRM4 mRNA expression was suppressed by SRRM4 ASO in a dose-dependent manner (Fig. 4A). Cell viability was successfully decreased by transfecting AmNA_7174 and alternative splicing of *REST* was changed by the formation of REST mRNA expression (Figs. 4B and C). The band of sREST (281 bp) decreased, while that of REST (231 bp) increased in dose-dependent manner, but no change with AmNA_26 (Fig. 4C). The REST and sREST mRNA are produced from a single gene through alternative splicing, suggesting that REST mRNA expression by transfecting ASO was caused through changing *REST* alternative splicing as seen in SCLC cells (Fig. 1).

## Discussion

SRRM4 ASO successfully repressed SRRM4 mRNA in SCLC cell culture and suppressed tumor formation in vivo [3]. SCLC cell lines expressed decreased levels of tumor repressor REST and increased the expression of the REST isoform sREST [14]. Inhibition of REST activity is a crucial step in the carcinogenesis of a subgroup of SCLC [16]. Intratumoral heterogeneity of SCLC is thus caused by REST activity [26]. Several SCLC cells lack REST expression, but sREST expression contributes to the abnormal expression of several neuronal genes and the survival and growth of human tumors [26]. To address the anti-tumor effects by treatment with SRRM4 ASO, we assumed that the expression of the tumor suppressor REST through modification of alternative splicing reduced cell viability. Herein, we report that *REST* splicing changes occurred in SCLC cells after transfection of SRRM4 ASO. Treatment with SRRM4 ASO induced REST expression through alternative splicing, followed by the repression of the abnormal expression of several neuronal genes, such as neuropeptides that function in autocrine growth in SCLC [13, 14, 27]. Micro array analysis using H146 mRNA after SRRM4 ASO treatment showed that 9 of 10 RE1-controlled genes previously reported [28] were highly repressed (> twofold higher compared to negative control), in which five of 9 genes were statistically significant (p < 0.05) (Additional file 1: Table S1). Furthermore, exogenous reconstitution of REST in SCLC cells, which predominantly express sREST, successfully reduced cell viability. However the effects of SRRM4 repression was statistically significant difference at around -70% repression, suggesting the possibilities due to the lower transfection efficiency of plasmids and/or intratumoral heterogeneity of SCLC containing SRRM4-negative cell population [29]. Taken together, SRRM4 ASO exhibited anti-tumor effects by suppressing SRRM4 mRNA expression through the modification of alternative splicing from sREST to REST.

SCLC cell lines were previously classified into two subtypes; classic and variant subtypes. The “classic” subtypes of SCLC cells predominantly grow as spherical aggregates of floating cells with or without central necrosis, while the “variant” subtype grows as either a tightly or loosely adherent monolayer. Previous study showed that SRRM4 ASO effectively suppressed SRRM4 expression and induced anti-tumor effects against N417 cells [3]. N417 cells were categorized as “variant” subtype, while both of H146 and H209 cells as “classic” subtypes based on SCLC cell classification [30, 31]. According to the recent report, H146 and H209 were classified as SCLC-A (ASCL1-high; “classic”) of SCLC but not SCLC-N (NeuroD1-high; “variant”) depending on the key transcription factor expression [23]. A study using a genetically engineered mouse model of SCLC suggested that SCLC-A is a necessary precursor of SCLC-N [9, 32]. ASCL1 is implicated as a driver in initial oncogenesis and NeuroD1-high tumors differentiating from or being selected from ASCL1-high precursor. Invasive tumors demonstrated a NeuroD1-high phenotype resembling the variant type of SCLC subtypes [33]. Furthermore, each single SCLC cell line in culture shows the intratumoral heterogeneity [34] that may contain possibly different cell population containing SRRM4 mRNA-high and -low cells. SRRM4 mRNA-high cells and SRRM4 mRNA-low cells may show heterogeneous morphology and various growths and tumorigenicities [31]. In situ hybridization analysis of SRRM4 mRNA expression revealed that H146 cells seem higher expression than N417 (Additional file 1: Fig. S8A). Both cell lines seem to contain a small population of SRRM4-negative cells showing intratumoral heterogeneity, further N417 showed higher Ki-67 positive cells rather than H146 (Additional file 1: Fig. S8B). Patients with higher Ki67-positive value are better survival than those with low Ki67 after chemotherapy [35]. On based on our results, both cell lines (H146 and H209) significantly decreased cell viability by transfecting SRRM4 ASO, but the effect was less than that of N417 cells [3]. It is possible that this was due to the intratumoral heterogeneity of the SCLC cell lines [36] and tumor heterogeneity is an important factor leading to the failure of conventional cancer therapies [37, 38]. RT-PCR analysis showed that faint REST mRNA was detected compared to sREST mRNA in H146 and H209 cells, suggesting the existence of cell population expressing endogenous REST (Fig. 1B). Even single SCLC cell line such as H146 or H209 shows intratumoral heterogeneity that may contain SRRM4 mRNA-high and SRRM4 mRNA-low cell populations. SCLC cells are also reported to contain heterogenous cell populations containing Notch, which induces cell cycle arrest [39], suggesting mature cell population expressing SRRM4 arrests the cell cycle in immature cell population through Notch signaling. We previously demonstrated that N417 cells cultured on Matrigel induced the SRRM4 expression [21], however, the effects of Matrigel had almost no effect on H146 and H209, as previously reported [31]. The expression level of SRRM4 mRNA is variable even in SCLC patients [6] as well as in SCLC cell lines (Additional file 1: Fig. S2) due to intratumoral heterogeneity [3]. Furthermore previous report by other group quantified apoptosis of H146 cells transfected with REST expression plasmid [16]. They concluded similar results with us that -22% of H146 cells exhibited apoptosis by FACS analysis. The effective suppression of SRRM4 by SRRM4 ASO may depend on the cell type possibly due to the expression level of SRRM4. For future effective therapeutic application of SRRM4 ASO, we need to analyze in detail the population of SRRM4-expressing cells in each SCLC cell line for intratumoral heterogeneity.

Neuroendocrine PCa (NEPC) is an extremely aggressive type of PCa. NEPC (t-NEPC) may develop de novo or in patients treated with hormonal therapeutic medicine as a mechanism of resistance [40]. In t-NEPC resistant to current therapies used in advanced PCa, high proliferative tumor dissemination can occur quite rapidly [41]. SRRM4, the RNA splicing factor of *REST*, abnormally drives the progression of NEPC and may be an effective therapeutic target [18, 42–44]. SRRM4 expression is associated with tumor progression and development of resistance against clinical treatments [45]. Using VCaP and 22Rv1 cells, PCa cell lines expressing SRRM4, SRRM4 ASO successfully reduced SRRM4 expression and cell viability in a dose-dependent manner through changing *REST* splicing similar to that in SCLC cells (Figs. 1 and 4). Thus, SRRM4 ASO might be a good therapeutic medicine for aggressive recalcitrant tumors expressing SRRM4. Recently, a study reported that SRRM4 plays a role in producing novel circular RNA by regulating microexon inclusion, and indicated high expression of SRRM4 in HeLa S3 cells [46]. Our preliminary analysis using RT-qPCR showed that low but significant levels of SRRM4 mRNA were expressed in HeLa S3 cells, and the transfection of SRRM4 ASO caused induced caspase3/7 expression. A recent study showed that InP/ZnS quantum dot treatment induced apoptosis, resulting in the downregulation of SRRM4 mRNA in HeLa cells, which prevented tumor growth [47]. Caspase activation caused by chemically-modified phosphorothioate ASO was previously reported, which correlates with the hepatotoxicity in vivo [48]. Our preliminary data has revealed that AmNA_7174 compared to AmNA_26 showed > 94% of genes were unaffected statistically by exon array analysis using H146 cells. Because AmNA_7174 specifically targets to SRRM4 mRNA, we searched the gene targets by AmNA_7174 with even 1 or 2 base mismatches using GGGenome database (gggenome.dbcls.jp). Of 280 genes by database search, 269 genes were confirmed on microarray ChiP. As a result, expression of 10 of the 269 genes were changed by AmNA_7174 (Additional file 1: Table S1). Although further detailed studies are needed, we are currently analyzing comprehensive gene expression for possible off-target effects and optimizing the structure of ASO for higher safety to develop future effective therapeutic medicine. During our study, we tested the specificity of anti-SRRM4 antibodies currently commercially available, however we found that all antibodies tested did not specifically recognize SRRM4 protein. We are also planning to produce anti-SRRM4 antibody for future study.

In summary, our developed SRRM4 ASO successfully reduced SRRM4 mRNA in SCLC and PCa cells and reduced cell viability. Repression of SRRM4 mRNA induced modified alternative splicing producing REST, followed by anti-tumor effects. The degree of anti-tumor effects depending on the subtypes of SCLC cells suggested that studies with appropriate biomarkers categorizing each subtype are important. Several clinical trials with various therapeutic targets appear to differ in subtype specificity. We are currently studying several subtypes of cells for the evaluation of SRRM4 ASO as well as companion diagnostics for future clinical trials.

## Conclusion

Our study demonstrated that administration of SRRM4 ASO causes the changes in the alternative splicing of *REST* through SRRM4 mRNA repression. Alternative splicing of *REST* from sREST to REST might be a potential cause of anti-tumor effects by SRRM4 ASO in neuroendocrine tumors SCLC as well as PCa.

## Supplementary Information

**Additional file 1**: **Figure S1**. Structure of Amido-bridged nucleic acid (AmNA) and LNA. **Figure S2**. SRRM4 expression in SCLC and PCa cells. **Figure S3**. Alternative splicing of *REST* by SRRM4. **Figure S4**. Cell viability analysis of H146 and H209 cells transfected with SRRM4 ASO. **Figure S5**. Cell viability analysis of A549 cells by transfecting SRRM4 ASO. **Figure S6**. Original image of western blot. **Figure S7**. Expression of SRRM4 mRNA in prostate cancer (PCa) cell lines. **Figure S8**. SRRM4 expression in SCLC lines (H146 and N417 cells). **Table S1**. REST-regulated genes decreased after transfecting SRRM4 ASO by microarray analysis

## Data Availability

Datasets used and/or analyzed during the current study are available from the corresponding author on reasonable request.

## Abbreviations

ASO: Antisense oligonucleotide
SRRM4: Ser/Arg repetitive matrix 4
SCLC: Small cell lung cancer
REST: RE1-silencing transcription factor
sREST: Splicing form of REST
PCa: Prostate cancer
NRSF: Neuron-restrictive silencer factor
AmNA: Amido-bridged nucleic acid
NSCLC: Non-SCLC
RT-qPCR: Reverse transcription-quantitative real time PCR
RT-PCR: Reverse transcription-PCR
RIN: RNA integrity number
RIPA: Radio-Immunoprecipitation assay
