# Supplementary material for: An antisense amido-bridged nucleic acid gapmer oligonucleotide targeting SRRM4 alters REST splicing and exhibits anti-tumor effects in small cell lung cancer and prostate cancer cells
Source: Cancer Cell Int. 2023 Jan 17;23:8. doi: 10.1186/s12935-022-02842-1 (PMC9847160; doi:10.1186/s12935-022-02842-1)
Supplement: Supplementary file 1 — Additional file 1: Figure S1. Structure of Amido-bridged nucleic acid (AmNA) and LNA. Figure S2. SRRM4 expression in SCLC and PCa cells. Figure S3. Alternative splicing of REST by SRRM4. Figure S4. Cell viability analysis of H146 and H209 cells transfected with SRRM4 ASO. Figure S5. Cell viability analysis of A549 cells by transfecting SRRM4 ASO. Figure S6. Original image of western blot. Figure S7. Expression of SRRM4 mRNA in prostate cancer (PCa) cell lines. Figure S8. SRRM4 expression in SCLC lines (H146 and N417 cells). Table S1. REST-regulated genes decreased after transfecting SRRM4 ASO by microarray analysis [file 12935_2022_2842_MOESM1_ESM.docx]

**Figure S1**

**
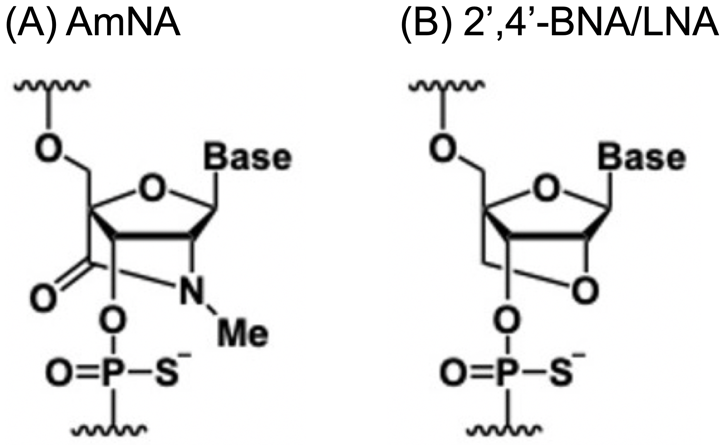
**

**Fig. S1.** Structure of Amido-bridged nucleic acid (AmNA) and LNA.

(A) amido-bridged nucleic acid (AmNA) (B) 2’-*O*,4’-*C*-methylene-bridged nucleic acid (2’,4’-BNA/LNA)

**Figure S2**


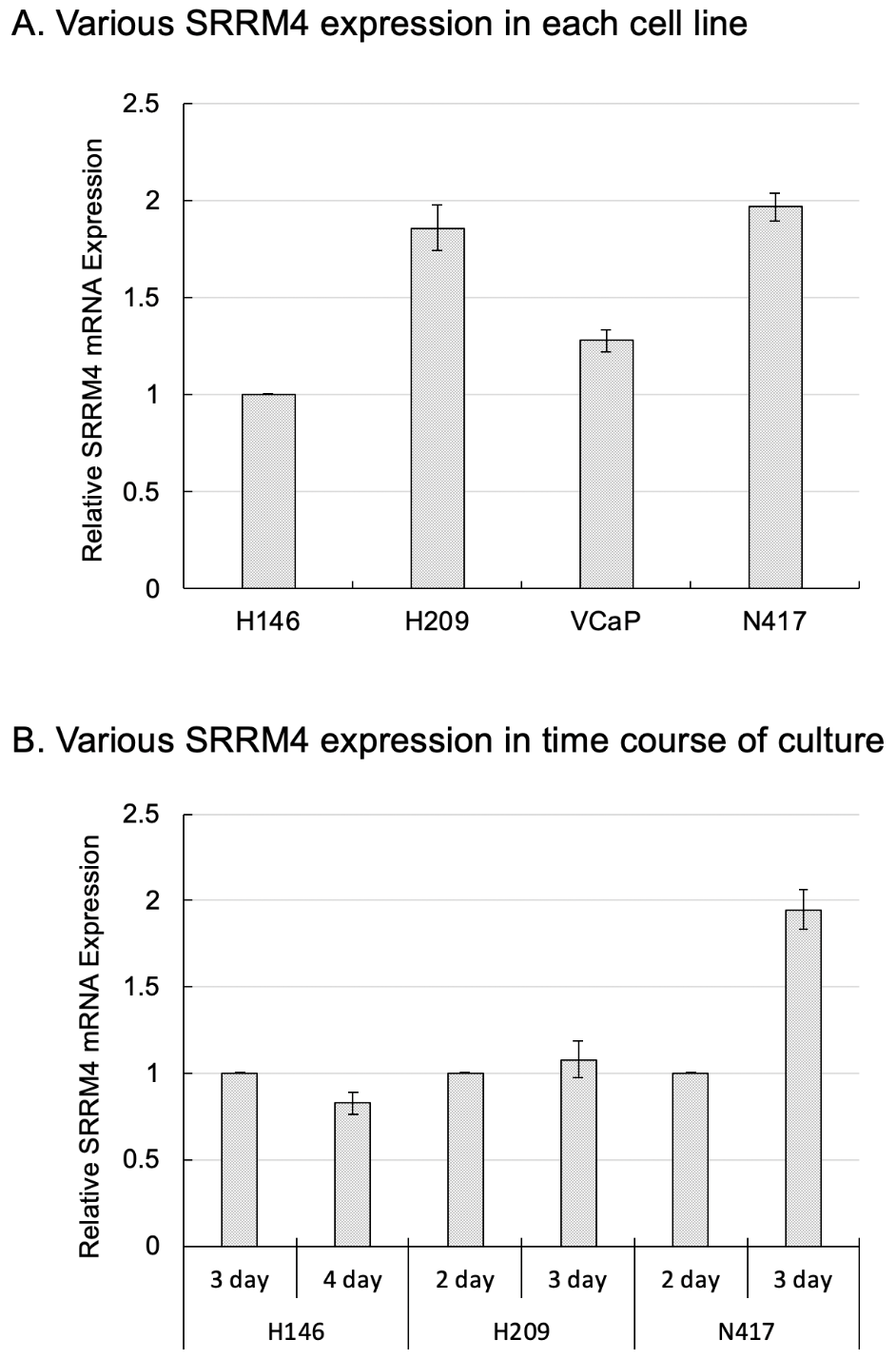


**Fig. S2.** SRRM4 mRNA expression in SCLC and PCa cells. SCLC cell lines (H146, H209 and N417 cells) and VCaP were analyzed for (a) SRRM4 mRNA expression and (b) SRRM4 mRNA expression in the time course of culturing.

**Figure S3**


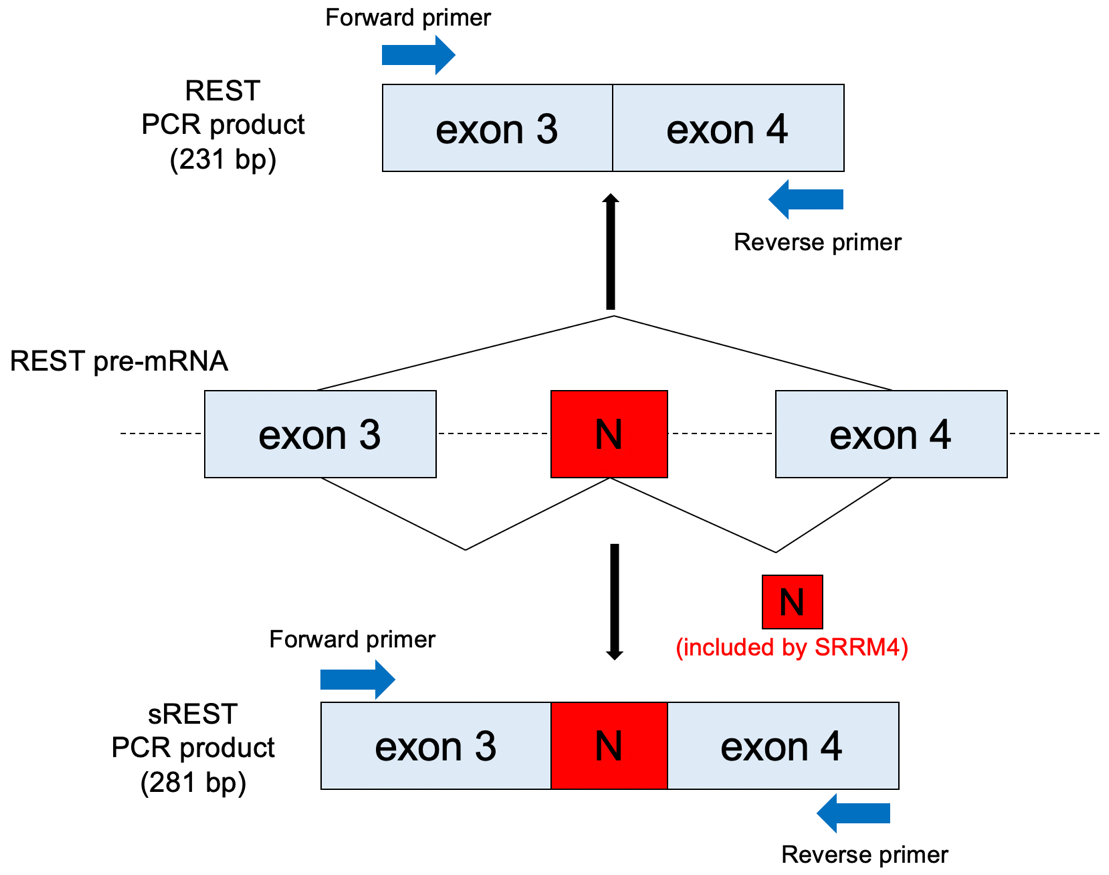


**Fig. S3.** Alternative splicing of *REST* by SRRM4. The splicing model of *REST* is shown. A REST isoform (sREST) is produced by SRRM4 incorporating the exon N between exons 3 and 4. Primers selected for RT-PCR are shown with blue arrows.

**Figure S4**


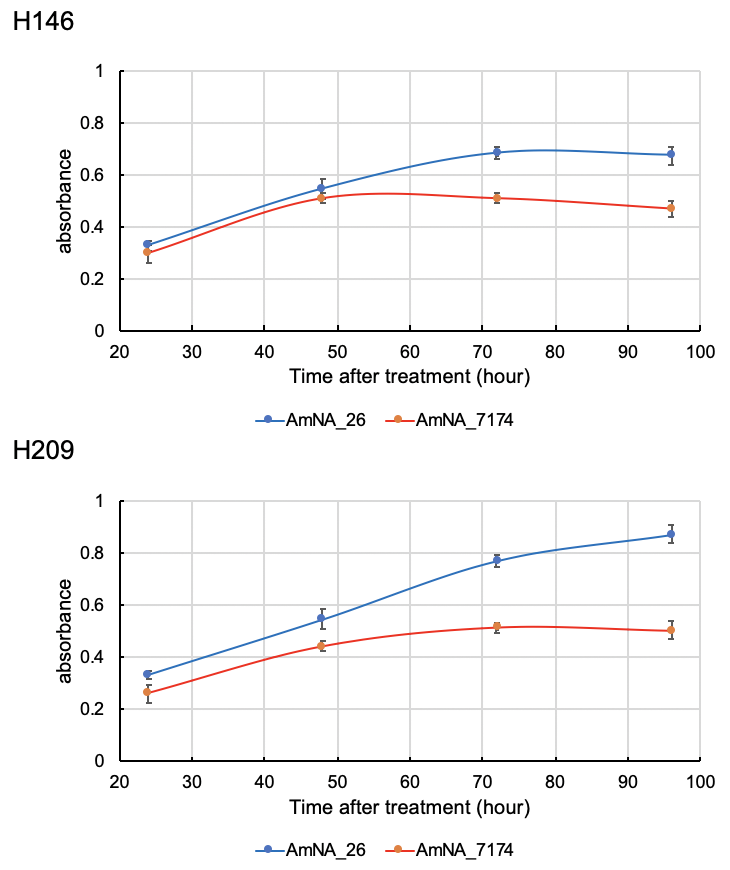


**Fig. S4.** Cell viability analysis of H146 and H209 cells transfected with SRRM4 ASO. SCLC cell lines (H146 and H209) were transfected with SRRM4 ASO and the cell viability assay was performed in the time course of culture.

**Figure S5**


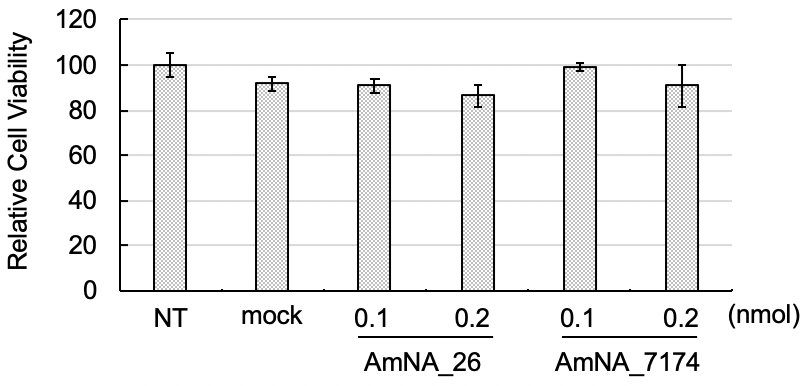


**Fig. S5.** Cell viability analysis of A549 cells by transfecting SRRM4 ASO. NSCLC cell lines (A549 cells) were transfected with SRRM4 ASO. Relative cell viability was not changed in this condition.

**Figure S6**


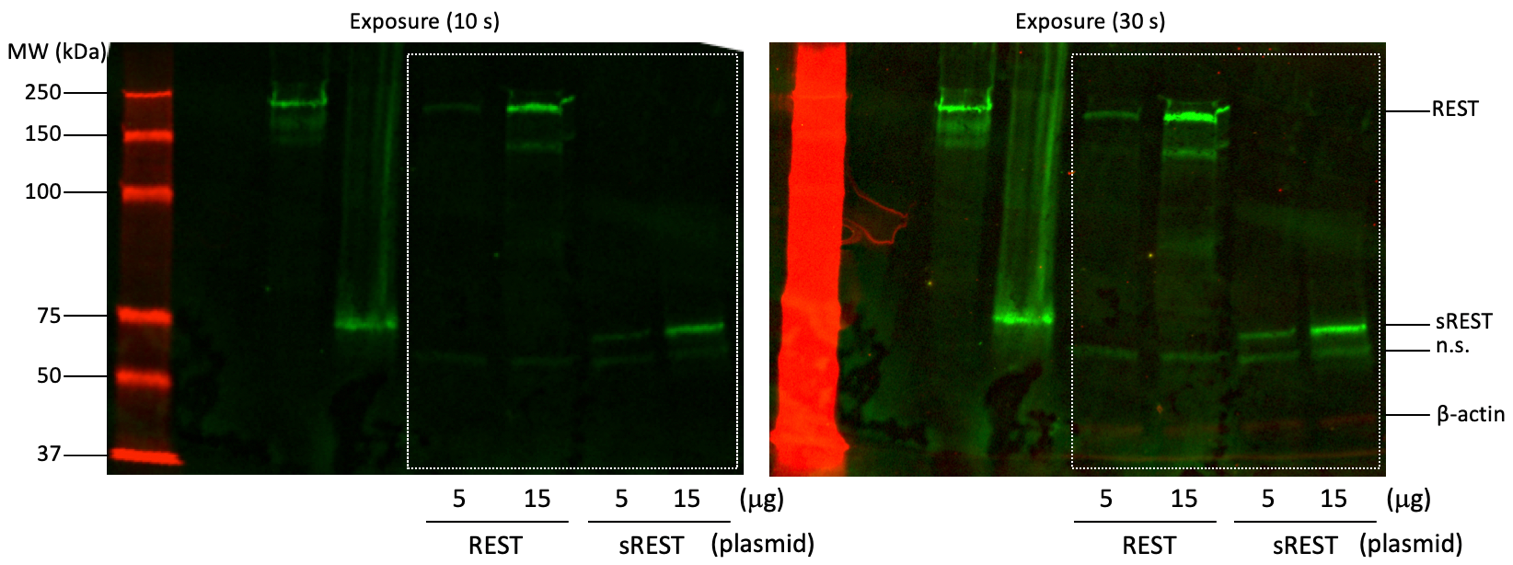


**Fig. S6.** Original image of western blot. The membrane was exposed for 10 s (left) and 30 s (right). The green and red band were images using anti-Flag and anti-actin antibodies, respectively. Molecular weight size marker was shown on left and each protein band was shown on the right. n.s.; non-specific band.

**Figure S7**


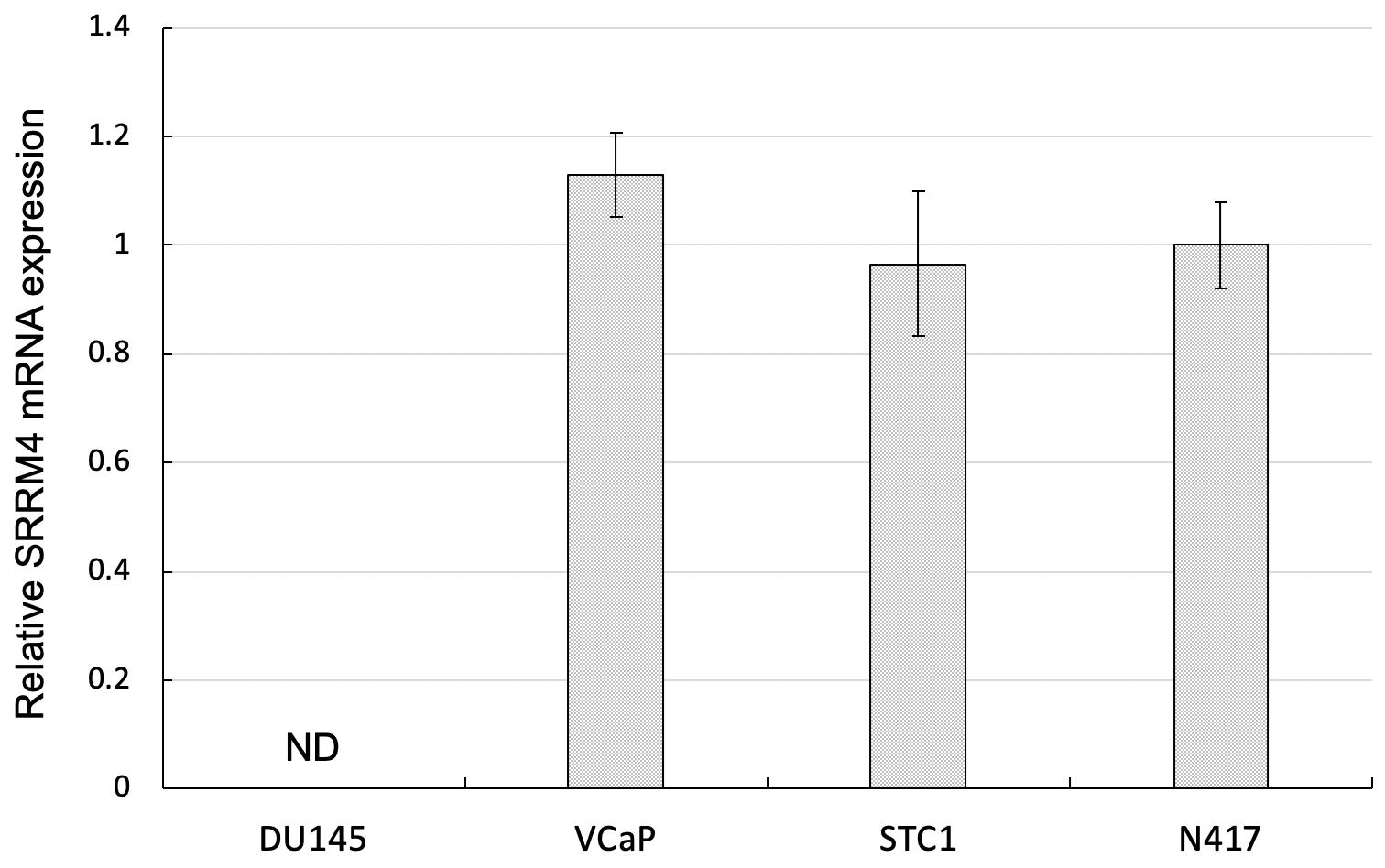


**Fig. S7.** Expression of SRRM4 mRNA in prostate cancer (PCa) cell lines. PCa (DU145 and VCaP) and SCLC cell lines (N417 and STC1) were analyzed by qRT-PCR. ND, not determined.

**Figure S8**


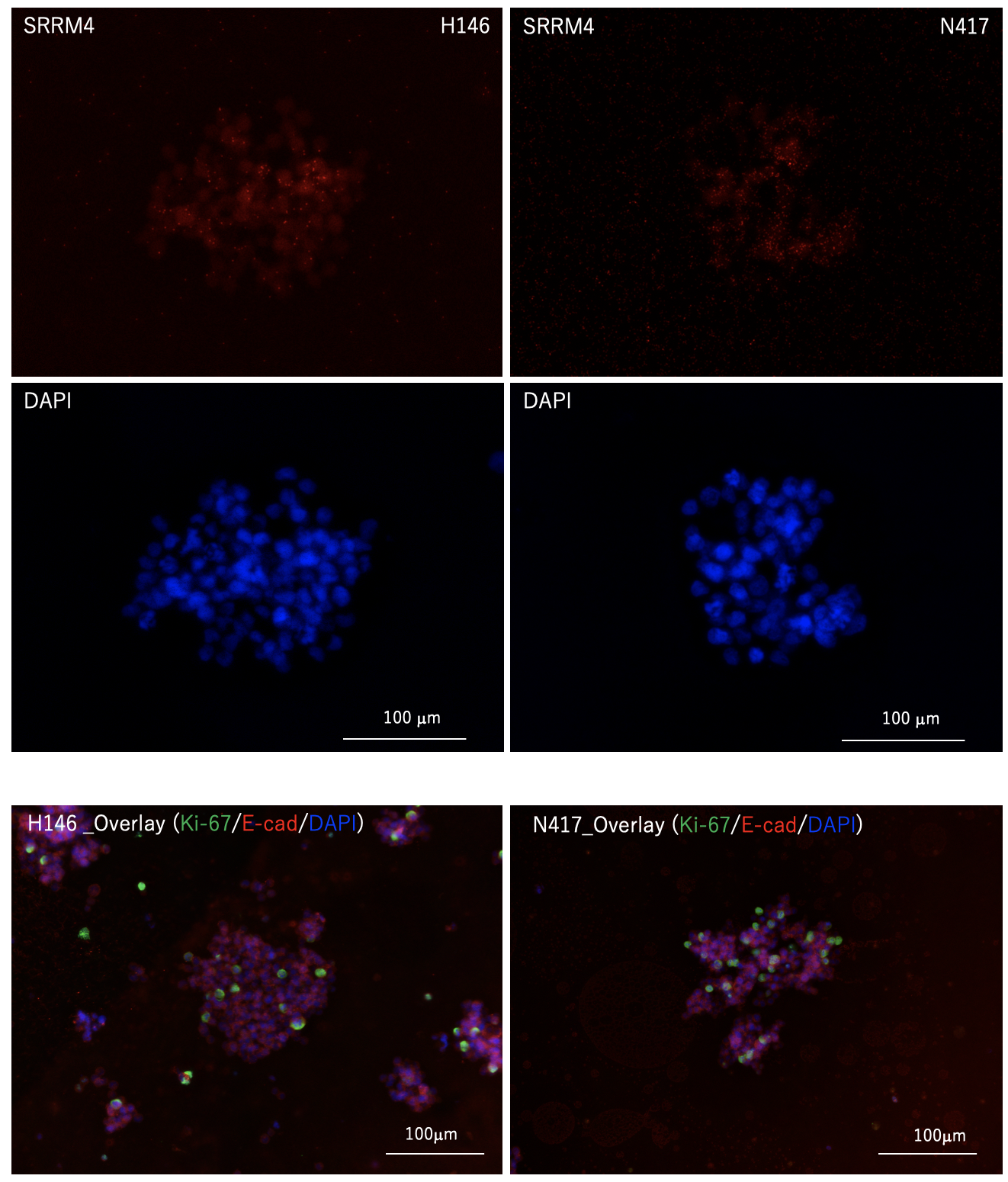


**Fig. S8.** SRRM4 expression in SCLC cell lines (H146 and N417 cells) . Expression of SRRM4 was analyzed by in situ hybridization and DAPI staining. Expression of Ki-67 was analyzed using anti-Ki67 antibody and anti-E-cadherin antibody. Images were overlaid of Ki67, E-cadherin and DAPI staining.

**Table S1**

| Gene Symbol | Fold Change | p-value |
| --- | --- | --- |
| INA | -12.89 | 9.04E-09 |
| CRMP1 | -2.72 | 0.0007 |
| TSC2 | -2.23 | 0.0027 |
| CELSR3; MIR4793 | -1.98 | 0.0065 |
| BEX1 | -1.51 | 0.0133 |
| RUNDC3A | -1.61 | 0.1037 |
| C3orf14 | -1.55 | 0.3145 |
| CHGB | -1.21 | 0.4916 |
| GLA | -1.07 | 0.0679 |
| APLP1 | 1.03 | 0.6697 |

**Table S1.** REST-regulated genes decreased after transfecting SRRM4 ASO (AmNA_7170) by Micro array analysis.　AmNA_7170 is four-bases different from AmNA_7174, that contains similar SRRM4 mRNA repression activity. Among of 10 REST-regulated genes reported in HEK293, 9 genes were significantly repressed after transfecting AmNA_7170 in H146 cells. The represssion could be effects of induction of REST mRNA expression through *REST* splicing.
